# Kiwifruit protease Act d 1 compromises the intestinal barrier by disrupting tight junctions

**DOI:** 10.1186/2045-7022-4-S2-O10

**Published:** 2014-03-17

**Authors:** Milica Grozdanovic, Milena Cavic, Uros Andjelkovic, Arnd Petersen, Joost Smit, Marija Gavrovic-Jankulovic

**Affiliations:** 1 Faculty of Chemistry, University of Belgrade, Department of Biochemistry, Belgrade, Serbia; 2 Institute for Oncology and Radiology of Serbia, Department of Experimental Oncology, Belgrade, Serbia; 3 Institute for Chemistry, Technology and Metallurgy, Department of Chemistry, Belgrade, Serbia; 4 Research Center Borstel, Division of Cellular and Molecular Allergology, Borstel, Germany; 5 Utrecht University, Institute for Risk Assessment Sciences, Utrecht, Netherlands

## Background

Actinidin (Act d 1) is a cysteine protease and major allergen of kiwifruit with diagnostic significance. It is the most abundant of the 11 kiwifruit allergens recognized and has been identified as a marker molecule of kiwifruit allergy. However, the mechanism underlining the oral route of exposure and sensitization to this allergen has yet to be elucidated. Working under the hypothesis that food proteases, as was shown for some inhalatory allergenic proteases, could reach the intestinal mucosa and surpass this barrier through proteolytic activity, we examined the following: Does Act d 1 have the ability to resist gastrointestinal digestion and reach the intestinal mucosa in a biologically active form? Upon reaching the intestinal mucosa does Act d 1 enhance permeability of the intestinal barrier by disrupting tight junctions?

## Method

*In vitro* analysis of Act d 1 digestion stability in simulated conditions of the gastrointestinal tract was performed by means of SDS-PAGE, zymography, ESI-TOF and immunoelectrophoresis. In addition, the influence of Act d 1 on tight junctions of Caco-2 cells was assessed by immunofluorescence and by measuring changes in transepithelial resistance and FITC-dextran leakage across cell monolayers. *In vivo* studies were performed to determine the effect of Act d 1 on intestinal permeability in mice.

## Results

Act d 1 isolated from kiwifruit under native conditions retained its primary structure, immunological reactivity and proteolytic activity after 2 h of simulated gastric digestion, followed by 2 h of simulated intestinal digestion. Exposure of confluent Caco-2 cells to Act d 1 reduced the transepithelial resistance of cell monolayers by 18.1% after 1h (P < 0.01) and 25.6% after 4 h (P < 0.001). The loss of barrier function was associated with leakage of FITC-dextran across the monolayers. Confocal microscopy revealed that Act d 1 treatment lead to disruption of tight junction proteins occludin and ZO-1. None of these effects were observed with heat inactivated Act d 1. *In vivo* measurements of intestinal permeability in mice showed that following administration of 40 kDa FITC-dextran by oral gavage, significantly higher concentrations of FITC-dextran (2.33 µg/mL) were later detected in the sera of Act d 1 treated mice in comparison to the control group (0.5 µg/mL, P < 0.05).

## Conclusion

Our findings show that Act d 1 is capable of reaching the intestinal mucosa in a proteolytically active and immunoreactive state and that it causes protease-dependent disruption of tight junctions in Caco-2 cells and induces intestinal permeability in mice. To the best of our knowledge, Act d 1 is the first food allergen whose proteolytic activity has been linked to breach of the epithelial barrier as a possible mechanism of sensitization.

